# Robotic Liver Surgery for Alveolar Echinococcosis: A Single-Centre Experience

**DOI:** 10.3390/pathogens11111276

**Published:** 2022-10-31

**Authors:** Kira C. Steinkraus, Laila Jötten, Benno Traub, Marin Zaimi, Maximilian Denzinger, Christoph W. Michalski, Marko Kornmann, Felix J. Hüttner

**Affiliations:** Department of General and Visceral Surgery, Ulm University Hospital, Albert-Einstein-Allee 23, 89081 Ulm, Germany

**Keywords:** alveolar echinococcosis, echinococcus multilocularis, robotic liver surgery, major liver resection

## Abstract

Alveolar echinococcosis (AE) is a rare disease caused by Echinococcosis multilocularis, which usually requires multidisciplinary management including surgery as the only curative approach. In recent years, minimally invasive strategies have been increasingly adopted for liver surgery. In particular, robotic surgery enables surgeons to perform even complex liver resections using a minimally invasive approach. However, there are only a few reports on robotic liver surgery for AE. Consecutive patients undergoing robotic liver surgery for AE were analysed based on the prospective database of the Interdisciplinary Robotic Centre of Ulm University Hospital. Between January 2021 and August 2022, a total of 16 patients with AE underwent robotic hepatectomy at our institution. Median age was 55.5 years (23–73), median body mass index (BMI) was 25.8 kg/m^2^ (20.2–36.8) and 12 patients (75%) were female. Anatomic resections were performed in 14 patients (87.5%), of which 4 patients (25%) underwent major hepatectomies (i.e., resection of >3 segments) including two right hemihepatectomies, one left hemihepatectomy and one extended right hemihepatectomy performed as associating liver partition with portal vein ligation staged (ALPPS) hepatectomy. There was no 90-day mortality, no postoperative bile leakage and no posthepatectomy haemorrhage. One patient developed posthepatectomy liver failure grade B after extended right hemihepatectomy using an ALPPS approach. One patient had to be converted to open surgery and developed an organ-space surgical site infection, for which he was re-admitted and underwent intravenous antibiotic therapy. Median length of postoperative hospital stay was 7 days (4–30). To our knowledge, this is the largest series of robotic liver surgeries for AE. The robotic approach seems safe with promising short-term outcomes in this selected cohort for both minor as well as major resections.

## 1. Introduction

Alveolar echinococcosis (AE) is considered a rare disease caused by the larval stage of Echinococcus multilocularis, also referred to as the fox tapeworm, which belongs to the family of Taeniidae [1]. It is described as a serious parasitosis in the Northern hemisphere, including Europe, parts of North and Central Asia, especially Japan, and North America, with great importance in the medical field due to its organ manifestation, which is commonly in the liver (75–98%) [2,3,4,5,6,7]. It is associated with a poor prognosis because of its infiltrative and sometimes metastatic growth, which resembles the characteristics of a malignant tumour [8,9]. Humans can be infected as accidental hosts by oral intake of viable eggs [6,10]. The early stages of AE may be asymptomatic for up to 15 years, leading to late and frequently incidental diagnosis of the disease. Clinical symptoms start to appear depending on the location and size of the lesions. Vesicles surrounded by large granulomas increase in diameter, with sizes greater than 10 cm or cystic liver occupation of more than 70% and may lead to symptoms by compression or infiltration of surrounding tissues or, for example, hepatic vessels or bile ducts [11,12,13,14]. According to the World Health Organization (WHO), echinococcosis in humans is among the 17 neglected tropical diseases [15,16]. To date, there are few studies regarding the nonmonetary burden and cost of AE. Torgerson et al. estimated that there are 18,200 new AE cases in China each year and calculated the nonmonetary burden using DALY (disability-adjusted life years) based on these numbers to be a median of 666,434 DALYs lost per year [17]. A recent study conducted by Lötsch et al. calculated the annual cost of AE to be EUR 680,000 in Austria alone [18].

Medical therapy with benzimidazoles, in particular albendazole, has substantially improved long-term survival, which had been <10% after ten years before the introduction of benzimidazoles [6,19,20,21,22]. However, the only curative therapeutic approach is radical surgery combined with adjuvant medical treatment. As radiological imaging keeps improving, AE tends to be incidentally diagnosed more frequently at an earlier stage, where patients can be cured by minor hepatic resections [12,23]. However, particularly in cases with an asymptomatic course the diagnosis is still often delayed, when the hepatic lesions are already advanced in size. Together with the infiltrative growth of the lesion, this leads to the necessity of complex and major liver resections in a substantial proportion of cases.

Although laparoscopic liver surgery has evolved as a therapeutic approach for a variety of liver diseases, the aforementioned complexity of the procedures has inhibited its widespread implementation in the treatment of AE. In a recent study conducted by Gloor et al., laparoscopic hepatectomy for AE was performed in 23 patients with 9% major liver resections, which turned out to be feasible and safe for AE patients with PNM stage 1 [24]. They additionally included 70 patients, who received open hepatectomy in contrast with a 61% rate of major liver resections. Overall, the laparoscopic approach was linked to shorter operation times, shorter hospital stays and lower major complication rates. Another study by Wan et al. published similar results, with 13 patients receiving laparoscopic liver surgery for AE, stating a major complication rate of 7.7% [25]. Robotic surgery has the potential to overcome the challenges of conventional laparoscopic surgery and thus may enable surgeons to transfer the benefits of minimally invasive surgery to patients with AE requiring such complex operations. However, due to the rarity of the disease, the current literature consists mainly of case reports regarding robotic surgery for AE. Therefore, the aim of the current study was to assess the feasibility, safety and efficacy of robotic surgery for AE in a cohort of consecutive patients at a specialized centre.

## 2. Results

### 2.1. Patients’ Baseline Characteristics

From January 2021 until August 2022, a total of 28 patients underwent surgery for AE at the Department of General and Visceral Surgery of Ulm University Hospital. A primary robotic approach was performed in 16 of these 28 patients (57.1%), whereas the remaining 12 patients (42.9%) received open surgery. The median age was 55.5 years (23–73) and the median BMI was 25.8 (20.2–36.8). Six patients (37.5%) had a bilobar disease and a median of two segments (1–4) were involved. Fourteen patients (87.5%) had IgG-enzyme-linked immunosorbent assay (ELISA)-positive serology with a median of 99 U/mL (24–264). Further demographic parameters are shown in Table 1.

### 2.2. Surgical Details

**Figure 1:**
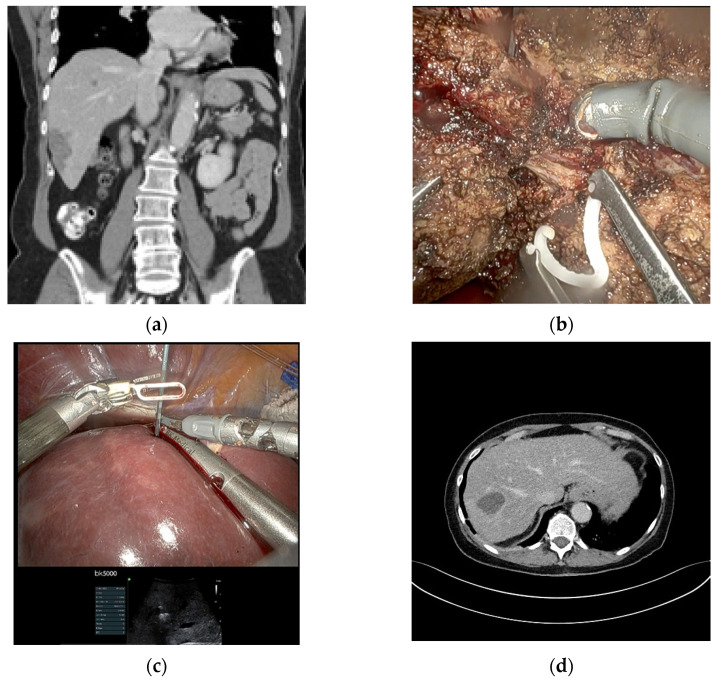
Patient with an AE lesion in S VI and a second small lesion in S VIII, who underwent anatomic S VI resection + radiofrequency ablation of the lesion in S VIII. (**a**) Preoperative CT scan (coronal view); (**b**) Intraoperative image showing the central ligation of the segmental portal vein branch to S VI; (**c**) Intraoperative image showing the ultrasound-guided radiofrequency ablation of the lesion in S VIII; (**d**) Postoperative CT scan showing the ablation result (transversal view).

**Figure 2:**
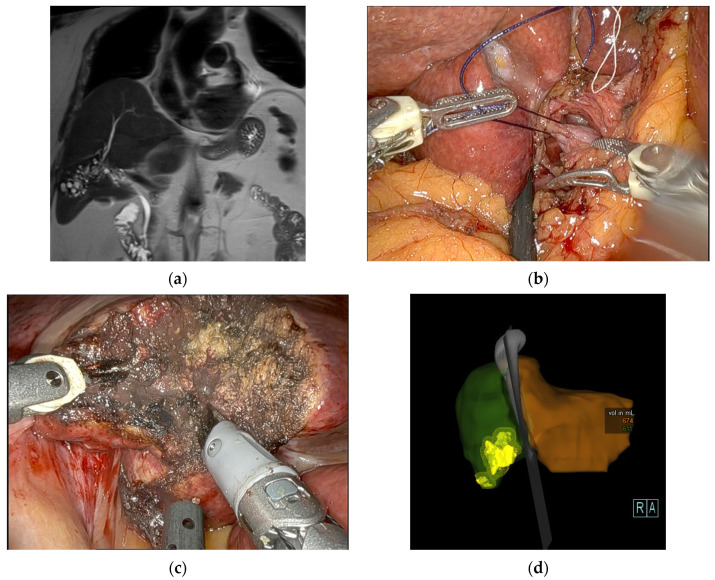
Patient with an AE lesion involving segments IVb/V/VIII with close contact to the right portal vein, who underwent an extended right hemihepatectomy by the ALPPS approach. (**a**) Preoperative MRI (T2-weighted coronal view); (**b**) Intraoperative image showing ligation of the right portal vein during the 1st step of ALPPS; (**c**) Intraoperative image showing the parenchymal dissection in S Ivb during the 1st step ALPPS; (**d**) CT liver volumetry 8 days after the 1st step ALPPS.

**Figure 3:**
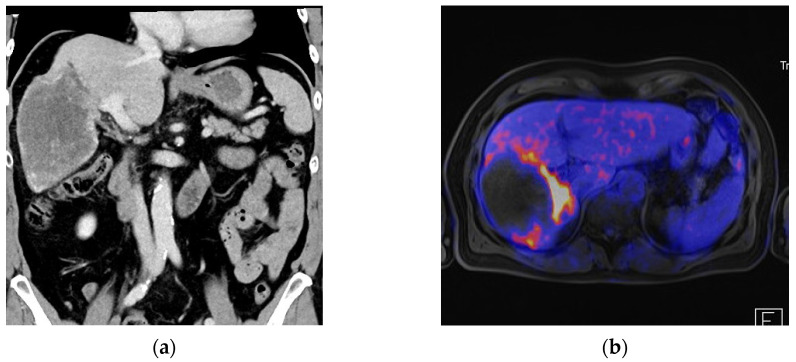

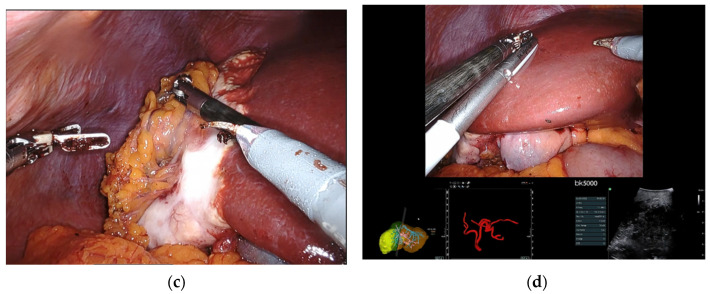
Patient with an AE lesion involving liver segments V/VI/VII/VIII with close contact to the right portal vein and the caval vein, who underwent a right hemihepatectomy including an atypical resection of S IVb. (**a**) Preoperative CT scan (coronal view); (**b**) Preoperative PET-MRI (transversal view) demonstrating activity of the lesion in close proximity to the portal vein; (**c**) Intraoperative image showing the large AE lesion in the right liver lobe; (**d**) Intraoperative image showing intraoperative resection planning with 3D-reconstructed CT models and intraoperative sonography.

Fourteen patients (87.5%) underwent anatomic resections, including four (25%) major hepatectomies, whereas two patients (12.5%) only received non-anatomical resections. The major hepatectomies consisted of two right hemihepatectomies, one left hemihepatectomy and one extended right hemihepatectomy performed in a robotic ALPPS approach. Both stages of the ALPPS procedure were carried out minimally invasively using a robotic approach with an interval of 13 days between stages 1 and 2. In terms of a modified ALPPS, the liver partition during the first stage was incomplete. There were two multivisceral resections, one simultaneous distal pancreatectomy due to an insulinoma and one simultaneous gastric wedge resection and partial resection of the diaphragm due to the extension of the AE lesion in the left liver lobe. There was one conversion to open surgery in a patient undergoing a right hemihepatectomy due to the large size of the lesion (12 cm) and close contact to the portal and caval veins. A detailed description of the surgical procedures is listed in Table 2. Median duration of surgery was 251 min (160–395) and the median estimated blood loss was 300 mL (100–1500). Only two patients (12.5%) received perioperative blood transfusions. Figure 1, Figure 2 and Figure 3 show examples of the preoperative work-up and intraoperative setting.

### 2.3. Postoperative Course

All patients were treated on the postoperative anaesthesia care unit after surgery and were transferred to the normal surgical ward on postoperative day (POD) 1. None of the patients had to be treated on the intensive care unit. The median postoperative hospital stay was 7 days (4–30). One patient was readmitted to the hospital 10 days after discharging due to an intra-abdominal fluid collection at the resection margin, classified as an organ/space surgical site infection (SSI), which was treated by intravenous antibiotic therapy with piperacillin/tazobactam (grade 2 complication). The patient undergoing an extended right hemihepatectomy by the robotic ALPPS approach developed PHLF grade B, which was treated with diuretics and albumin infusions. Furthermore, this patient developed a pleural effusion, which was treated by a one-time pleurocentesis, categorized as a grade 3a complication according to the Clavien–Dindo classification. The patient, who underwent a simultaneous distal pancreatectomy developed a biochemical leakage (POPF grade A). The median comprehensive complication index (CCI) was 0 (0–33.5) and the major complication rate grade ≥3 was 1/16 (6.1%). There was no postoperative mortality within 90 days. Detailed information on postoperative morbidity and mortality is shown in Table 3.

The margin negative (R0) resection rate was 12/16 (75%), whereas four patients (25%) had an R1 resection with a resection margin of <1 mm. The median follow-up was 134 days (5–365) and all patients were alive without any signs of recurrent disease at the last follow-up. One patient stopped postoperative albendazol therapy due to the development of a depression, which the patient associated with the antihelmintic therapy.

## 3. Discussion

In the current cohort, robotic surgery for AE of the liver proved to be feasible and safe for both minor as well as major hepatectomies. The majority of patients (87.5%) underwent anatomic liver resections and two patients received an additional intraoperative radiofrequency ablation. The postoperative morbidity was low and, particularly, there was no mortality and no cases of postoperative bile leakage or posthepatectomy haemorrhage. None of the patients had to undergo a reoperation. Postoperative morbidity consisted mainly of grade I/II complications with only one grade IIIa complication. Thus, the morbidity rates are in the same range as in previous reports on minimally invasive liver resection for AE [24,25] and even lower than in cohorts of open surgery for AE [24,26,27,28]. However, the lower complication rate in comparison to cohorts undergoing open liver resection for AE may be caused by a potential selection bias towards easier resections in the minimally invasive cases.

The R0 rate was in an acceptable range and comparable to other reports, even though WHO guidelines recommend a safety margin of 2 cm [12,24,28]. However, recent studies including one previous study from our centre have demonstrated that smaller resection margins (i.e., >1 mm) also demonstrate promising outcomes when antihelmintic therapy is continued postoperatively. Both Hillenbrand et al. and Gloor et al. state that no patient developed a recurrence during their follow-ups of 8.3 years and 55 months, respectively, even in margin-positive histopathology [24,29]. Nevertheless, within the current cohort, the postoperative follow-up is too short to discuss recurrence. 

Although minimally invasive surgery is increasing rapidly for a variety of indications, there are as yet only a few reports on minimally invasive surgery for hepatic AE [25,30,31]. On one hand, this might be caused by the rarity and regionality of the disease. On the other hand, this is probably attributable to the frequently challenging resections that are required for radical resection of AE lesions. Thus, there are few reports on laparoscopic surgery for AE on small cohorts from experienced centres [22,23,30]. 

Robotic surgery can overcome the challenges of conventional laparoscopic surgery due to its enhanced three-dimensional vision and the precision of the instruments, which can be angulated in any direction allowing accurate dissection also in difficult to reach areas. Nonetheless, the literature on robotic liver surgery for AE is scarce and mainly consists of case reports. Golriz et al. reported a case of right hemihepatectomy for AE in a 62-year-old male who developed an intraabdominal fluid collection postoperatively, which was drained percutaneously [32]. Similarly, Zhao et al. presented a case series of five patients with hepatic echinococcosis of the posterosuperior segments undergoing robotic surgery. However, only one of the patients had AE and underwent right hemihepatectomy with only a grade I postoperative complication, which was not further specified [31].

Thus, the current report represents the largest series on robotic surgery for AE with a variety of procedures, of which the majority (87.5%) were anatomic resections including four major hepatectomies. This demonstrates the versatility of the robotic approach in the treatment of AE, covering all hepatic segments and different types of resections. In the hands of experienced hepatobiliary surgeons, robotic surgery can be implemented quickly with safe outcomes. In our department, robotic surgery in general was implemented in October 2020 and the first hepatectomy for AE, a right hemihepatectomy, was performed in March 2021. After the implementation, it soon evolved to the primary approach for hepatic AE at our centre, whenever the lesions were deemed resectable by a robotic approach and did not present the need for vascular resection or extrahepatic involvement. In the current manuscript, there was no formal analysis of the difficulty of liver resections or learning curve, because this would not have been reasonable since robotic liver resection for other indications developed during the same time. However, it has been demonstrated by other authors that the learning curve for robotic hepatectomy can be rapidly overcome by surgeons with sufficient experience in laparoscopic surgery [33]. The current report corroborates that even under the above-mentioned circumstances, a robotic liver surgery program can be implemented safely, which has also been shown by other institutions [34]. However, adequate pre- and intraoperative planning of the resections is key to achieve good outcomes, because haptic feedback is missing in robotic surgery. In the preoperative setting, three-dimensional reconstructions of the major vasculature of the liver can help to prepare for complex resections. These 3D models can also be displayed during surgery on the surgeon’s console to aid the surgeon during the resection [35]. In the future, an augmented reality overlay would be preferable, but this is not yet routinely feasible. To finally define the necessary extent of the resection, an intraoperative sonography is obligatory during robotic hepatectomy. Furthermore, the intraoperative sonography, which can also be projected into the surgeon console, can help to clarify the proximity of major vessels to the lesion that is to be resected [36].

Although the current results are very promising, they have to be interpreted in the light of the limitations of the current report. The main limitation is that this was a retrospective study of a selected cohort of AE patients without a control group. On the other hand, the results are based on a prospectively managed database including prospective digital acquisition of morbidity and mortality data. Furthermore, all consecutive patients that underwent surgery by a primary robotic approach were included and the selection of the full cohort of AE patients is clearly described.

However, taken together, the results of our study suggest promising short-term outcomes for the continuous adoption of robotic resection for AE into the rapidly expanding field of robotic surgery.

## 4. Materials and Methods

### 4.1. Patient Selection and Preoperative Management

All adult patients undergoing robotic hepatectomy for AE at the Department of General and Visceral Surgery of Ulm University Hospital between January 2021 and August 2022 were identified from the prospective database of the Interdisciplinary Robotic Centre of Ulm University Hospital. The database is registered in the German Clinical Trials Register (DRKS00024946) and the local ethics committee of the University Ulm approved the study (137/21). An informed consent was obtained from all included patients. Ulm University Hospital is a tertiary care hospital and a supraregional centre for AE. Inclusion criteria were resectable hepatic lesions of AE and an ASA grade ≤III. Exclusion criteria were invasion of critical vessels of the hepatic hilum or liver veins, extra-hepatic metastasis of AE and liver cirrhosis of Child–Pugh grades B or C. All cases were individually presented and discussed within a multidisciplinary board, including specialists in infectiology, microbiology, radiology, nuclear medicine and surgery, in order to allow multidisciplinary decision making for optimal patient care. The diagnostic workup included abdominal ultrasound (US), computed tomography (CT), magnetic resonance imaging (MRI) and/or positron emission tomography (PET)-CT or PET-MRI and serological tests (IgG ELISA). The classification of AE was performed according to the WHO case definition. Treatment decisions were based on the recommendations of the WHO’s Informal Working Groups on Echinococcosis (WHO-IWGE). 

All included patients received antihydatid therapy with albendazole as a perioperative prophylaxis. Albendazole therapy was resumed after surgery as soon as aspartate aminotransferase (AST) and alanine aminotransferase (ALT) values returned to the normal range. Therapy continuation was recommended for two years postoperatively in all patients, even after R0 resection. After surgery, patients were followed-up regularly at the outpatient clinic of the Division of Infectious Diseases every 3 to 6 months.

### 4.2. Surgical Procedure

**Figure 4:**
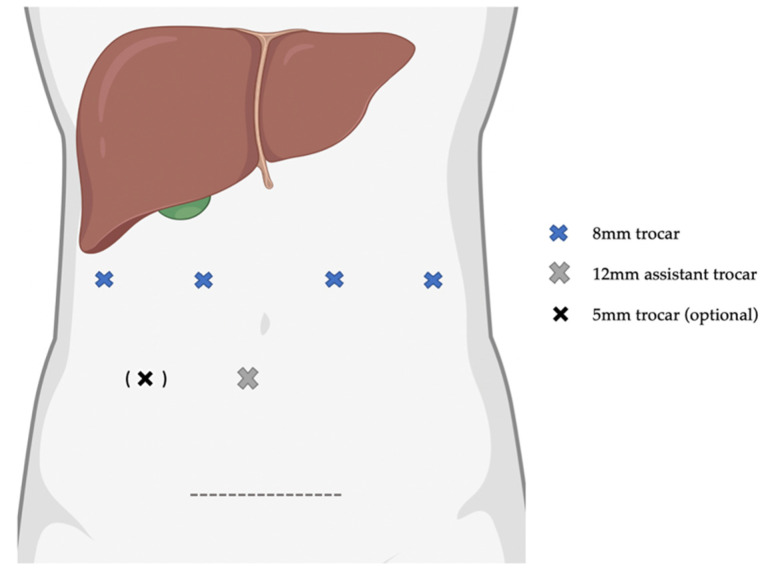
Schematic diagram of individual trocar placements in robotic AE liver resection. Four 8mm robotic trocars placed on a horizontal line approximately 2 cm above the umbilicus with a distance of 8 cm between each trocar. A 12 mm assistant trocar was placed triangulated below trocars 2 and 3. An optional 5 mm trocar was placed in major hepatectomies triangulated below trocars 1 and 2.

Surgery was performed using the DaVinci Xi Surgical System^®^ (Intuitive Surgical Inc., Sunnyvale, CA, USA). Patients were placed in the supine position with the legs spread apart. Usually, a slight anti-Trendelenburg position of 10–15° and a slight tilt to the left of 5–10° was established. The pneumoperitoneum was created by introducing a Veress needle at the subcostal margin in the left midclavicular line (Palmer’s point) with a pressure of 12–14 mmHg. Four 8 mm robotic trocars were placed on a horizontal line approximately 2 fingers above the umbilicus with a distance of approximately 8 cm between the trocars. The camera port (trocar 2) was usually placed at the level of the inferior vena cava. One trocar was placed further on the patient’s right side (surgeon’s left hand) and two on the patients’ left side (surgeon’s right hand). Furthermore, one 12 mm assistant trocar was placed triangulated below trocar 2 and 3, and in some cases, especially major hepatectomies, another 5 mm trocar was placed triangulated below trocar 1 and 2, shown in Figure 4. A laparoscopic, diagnostic exploration of the abdominal cavity was performed in all cases to exclude undetected extrahepatic disease or other intraabdominal pathologies. Intraoperative sonography was performed in all cases to compensate for the lack of haptic feedback and to determine the resection plane. Indocyanine green fluorescence imaging was performed in some cases to identify the major vasculature of the liver, biliary structures or the demarcation line after extrahepatic inflow control. For all major resections, an extrahepatic Glissonean approach was used. Furthermore, for all major resections, liver volumetry of the future liver remnant and 3D reconstructions of the hepatic arteries, portal vein and liver veins were performed, which were intraoperatively displayed onto the surgeon console. Parenchymal transection was performed by a combination of bipolar forceps and monopolar scissors or with a SynchroSeal device (Intuitive Surgical Inc., Sunnyvale, CA, USA). For hemihepatectomies and left lateral sectionectomies, a laparoscopic ultrasonic surgical aspirator was additionally used, which was guided by the table surgeon. After complete resection, the specimen was placed in an extraction bag and retrieved via a Pfannenstiel incision. In some patients with previous surgery, the old laparotomy site was used as the extraction site. Haemostasis was achieved by bipolar cautery and/or sutures. Neither sealants nor drains were used routinely and were only placed in selected cases at the discretion of the operating surgeon. For patients with additional small lesions located deep in the liver parenchyma, the possibility of simultaneous radiofrequency ablation during robotic surgery was evaluated in a parenchyma-sparing approach. Postoperative management was performed according to fast-track principles including early mobilization of the patient and early oral nutrition starting on POD 1. 

### 4.3. Clinical Definitions

The type of liver resection was classified according to the Brisbane 2000 system [37]. All resections that included complete resection of at least one liver segment were classified as anatomic resections, even if further non-anatomical resections or non-anatomical extensions were performed during the same procedure. Major hepatectomy was defined as resection of ≥3 liver segments.

The Clavien–Dindo classification was used to grade postoperative complications [38]. Postoperative biliary leakage (BL) [39], posthepatectomy liver failure (PHLF) [40] and posthepatectomy haemorrhage [41] were defined according to the definitions of the International Study Group of Liver Surgery (ISGLS). Surgical site infections (SSI) were divided into superficial, deep and organ/space SSIs according to the definition of the Centres for Disease Control [42]. Organ/space SSIs include intraabdominal infected fluid collections or abscesses. Postoperative pancreatic fistula was defined according to the definition of the International Study Group of Pancreatic Surgery [43].

### 4.4. Statistical Analysis

A descriptive statistical analysis with applicable measures of the empirical distribution of all baseline characteristics and endpoints was performed. Medians and ranges were calculated in the case of continuous variables, and absolute and relative frequencies were calculated in the case of categorical data. The statistical analyses were conducted with the statistical software R (R Foundation for Statistical Computing, Vienna, Austria. http://www.R-project.org, accessed on 1 September 2022). 

## 5. Conclusions

Short-term outcomes suggest that robotic surgery for AE of the liver is feasible and safe with promising outcomes in the current cohort. Not only minor resections, but also major hepatectomies may be performed safely. To create more robust evidence on robotic surgery for AE, multicentric analyses, e.g., a multi-institutional registry, would be desirable.

## Figures and Tables

**Table 1:** Clinical baseline characteristics.

	N = 16
Median age in years (range)	55.5 (23–73)
Median BMI in kg/m^2^ (range)	25.8 (20.2–36.8)
Gender	
Female	12 (75%)
Male	4 (25%)
ASA * score	
I	1 (6.2%)
II	8 (50%)
III	7 (43.8%)
WHO classification (PNM ^†^)	
P1N0M0	4 (25%)
P2N0M0	5 (31.3%)
P2N1M0	2 (12.5%)
P3N0M0	4 (25%)
P4N0M0	1 (6.2%)
Distribution of affected liver lobes	
Left	5 (31.2%)
Right	5 (31.2%)
Both	6 (37.5%)
Distribution of affected liver segments	
II	1(6.2%)
II, III	2 (12.5%)
II, III, V	1 (6.2%)
II, III, V, VIII	1 (6.2%)
II, V	1 (6.2%)
II, III, IVa, IVb	1 (6.2%)
II, III, VIII	1 (6.2%)
III, IVb	1 (6.2%)
IVb, V, VIII	1 (6.2%)
V, VI, VII, VIII	1 (6.2%)
VI	1 (6.2%)
VI, VII	2 (12.5%)
II, III, VI und IV	1 (6.2%)
VI, VIII	1 (6.2%)
Number of Lesions	
1	10 (62.5%)
2	4 (25.0%)
3	1 (6.2%)
5	1 (6.2%)
Median size of the lesions in cm (range)	5.3 (2.0–12.0)
Median time period of preoperative albendazole therapy in years (range)	1.78 (0.1–9.0)

* American Society of Anaesthesiologists (ASA). ^†^ P = parasitic mass in the liver, N = involvement of neighbouring organs, M = metastasis (PNM).

**Table 2:** Surgical details.

	N = 16
Extent of resection	
Major (>3 segments)	4 (25%)
Minor (≤3 segments)	12 (75%)
Type of resection	
Anatomic	14 (87.5%)
Non-anatomic	2 (12.5%)
Multivisceral resection *	2 (12.5%)
Previous hepatic surgery	1 (6.2%)
Surgical procedure	
Right hemihepatectomy	2 (12.5%)
Extended right hemihepatectomy (ALPPS)	1 (6.2%)
Left hemihepatectomy	1 (6.2%)
Left lateral sectionectomy	3 (18.8%)
Anatomic segmentectomy ^†^	7 (43.8%)
Atypical resection ^‡^	2 (12.5%)
R-status	
R0	12 (75%)
R1 (<1 mm)	4 (25%)
Median duration of surgery in minutes (range) ^§^	251 (160–395)
Median estimated blood loss in millilitre (range) ^∥^	300 (100–1500)
Perioperative blood transfusions	2 (12.5%)

* 1× simultaneous distal pancreatectomy due to insulinoma together with two atypical resections (segments II and VIII); 1× simultaneous gastric wedge resection and partial resection of the diaphragm together with anatomic resection of segment II. ^†^ 1× additional radiofrequency ablation of segment VIII. ^‡^ 1× additional radiofrequency ablation of segment Iva. ^§^ n = 15, because the patient undergoing ALPPS is not included; duration of the 1st step was 110 min and of the 2nd step 339 min. ^∥^ n = 15, because the patient undergoing ALPPS is not included; estimated blood loss of the 1st step was 200 millilitres and of the 2nd step 1500 millilitres.

**Table 3:** Postoperative morbidity.

	N = 16
90-day mortality	0 (0%)
90-day morbidity	
Clavien–Dindo I–II	3 (18.8%)
Clavien–Dindo III–V	1 (6.2%)
Median comprehensive complication index (range)	0 (0–33.5)
Surgical site infection (SSI)	
Superficial incisional SSI	0 (0%)
Deep incisional SSI	0 (0%)
Organ/space SSI	1 (6.2%)
Postoperative bile leakage	0 (0%)
Posthepatectomy haemorrhage	0 (0%)
Posthepatectomy liver failure	
Grade A	0 (0%)
Grade B	1 (6.2%)
Grade C	0 (0%)
Pleural effusion	1 (6.2%)
Postoperative pancreatic fistula	
Biochemical leakage (Grade A) *	1 (6.2%)
Grade B	0 (0%)
Grade C	0 (0%)
Reintervention ^†^	1 (6.2%)
Reoperation	0 (0%)
Rehospitalization ^‡^	1 (6.2%)
Median length of postoperative hospital stay in days (range)	7 (4–30)

* in the patient undergoing simultaneous distal pancreatectomy. ^†^ pleurocentesis for pleural effusion. ^‡^ due to organ/space SSI requiring intravenous antibiotic treatment.

## Data Availability

Not applicable.
